# Development of novel miR-129 mimics with enhanced efficacy to eliminate chemoresistant colon cancer stem cells

**DOI:** 10.18632/oncotarget.22322

**Published:** 2017-11-06

**Authors:** Ning Wu, Andrew Fesler, Hua Liu, Jingfang Ju

**Affiliations:** ^1^ Department of Pathology, School of Medicine, Stony Brook University, Stony Brook, NY, USA; ^2^ Key Laboratory of Experimental Marine Biology, Institute of Oceanology, Chinese Academy of Sciences, Qingdao, China

**Keywords:** 5-fluorouracil, miR-129, colorectal cancer, cancer stem cells, chemoresistance

## Abstract

**Background:**

Resistance to 5-Fluorouracil (5-FU) based chemotherapy is the major reason for failure of treating patients with advanced colorectal cancer.

**Materials and methods:**

In this study, we developed a novel miR-129 mimic with potent efficacy in eliminating resistant colon cancer stem cells both *in vitro* and *in vivo*. We integrated 5-FU into miR-129 by replacing Uracil (U) to generate 5-FU-miR-129 mimics (Mimic-1).

**Results:**

Mimic-1 is a strong therapeutic candidate with a number of unique features. Mimic-1 can be delivered to cancer cells without any transfection reagents (e.g. lipids, viral vector, nanoparticles). Mimic-1 is more potent at inhibiting cell proliferation and inducing cell cycle arrest at G1 phase than native miR-129 and the other mimics tested, while retaining target specificity. Mimic-1 prevents colon cancer metastasis *in vivo* without toxicity.

**Conclusion:**

This represents a significant advancement in the development of a nontoxic and highly potent miRNA based cancer therapeutics and establishes a foundation for further developing Mimic-1 as a novel anti-cancer therapeutic for treating colorectal cancer.

## INTRODUCTION

Colorectal cancer ranks third among cancer types in the United States with over 140,000 new cases each year [1]. 5-Fluorouracil (5-FU) based chemotherapy (e.g. FOLFOX) has been the major treatment option for metastatic colorectal cancer for well over 50 years [2, 3]. Despite advancements in early detection and improved treatment strategies, resistance to 5-FU based chemotherapy remains one of the major reasons for the failure of colorectal cancer therapy in advanced stage patients. Although the resistance mechanism is quite complex involving elevated target protein thymidylate synthase (TS), *TP53* mutation/deletion, and DNA repair, it has now been well established that resistance is due, at least in part, to the presence of highly resistant colon cancer stem cells [4, 5]. These cells, which are highly plastic in nature due to regulation by epigenetic mechanisms such as miRNA, represent an important therapeutic target [6].

Over the past decade, small regulatory RNAs have gained enormous interests in colon cancer research. Nearly 97% of RNAs are non-coding and many of these have important regulatory functions. miRNAs are a class of non-coding RNA molecules, 18-25 nucleotides in length, that regulate the expression of their target genes by translational arrest or mRNA cleavage mostly via direct interaction with the 3’-UTRs of target mRNAs [7, 8]. Base pairing between at least six consecutive nucleotides within the 5’-seed region of the miRNA with the target site on the mRNA is reported to be a minimum requirement for miRNA-mRNA interaction [9]. miRNAs have been found to regulate many cellular processes including apoptosis [10–13], differentiation [8, 14, 15] and cell proliferation [10, 15–17].

miRNA based therapy may offer a unique advantage as it can target multiple targets and pathways making it more difficult for tumor cells to escape cell death. There are also challenges for miRNA based therapeutics such as stability and delivery to tumor cells. Tremendous amounts of research efforts have been devoted to overcome these issues [18]. In this study, we have developed a novel strategy to overcome these critical bottlenecks using some unique and novel miRNA modifications. Previous studies have shown that mir-129 suppresses expression of BCL2, TS, and E2F3 in colon cancer [19]. miR-129 expression is able to inhibit colon tumor growth *in vitro* and *in vivo* [19]. There is also a progressive loss of miR-129 expression in colon cancer disease progression as a result of epigenetic regulation [19, 20]. This data all supports the premise of a miR-129 based therapeutic for colon cancer [21]. Using miR-129 as a therapeutic miRNA candidate, we were able to engineer a series of miR-129 mimics to further enhance the therapeutic efficacy.

Our results show that the 5-FU-miR-129 mimic (Mimic-1) is the best therapeutic candidate as it has a number of unique features such as enhanced stability and efficacy. We demonstrated that Mimic-1 was able to retain target specificity with enhanced cell cycle arrest induction. More importantly, Mimic-1 was capable of effectively eliminating highly resistant colon cancer stem cells and inhibiting metastatic tumor formation *in vivo*. Another breakthrough feature is that we can deliver Mimic-1 without any delivery vehicle into cancer cells *in vitro*. As a result, Mimic-1 has great potential as an ideal candidate for future anti-cancer therapeutic development.

## RESULTS

### Development and characterization of novel miR-129 mimics with enhanced target efficacy and stability

**Figure 1:**
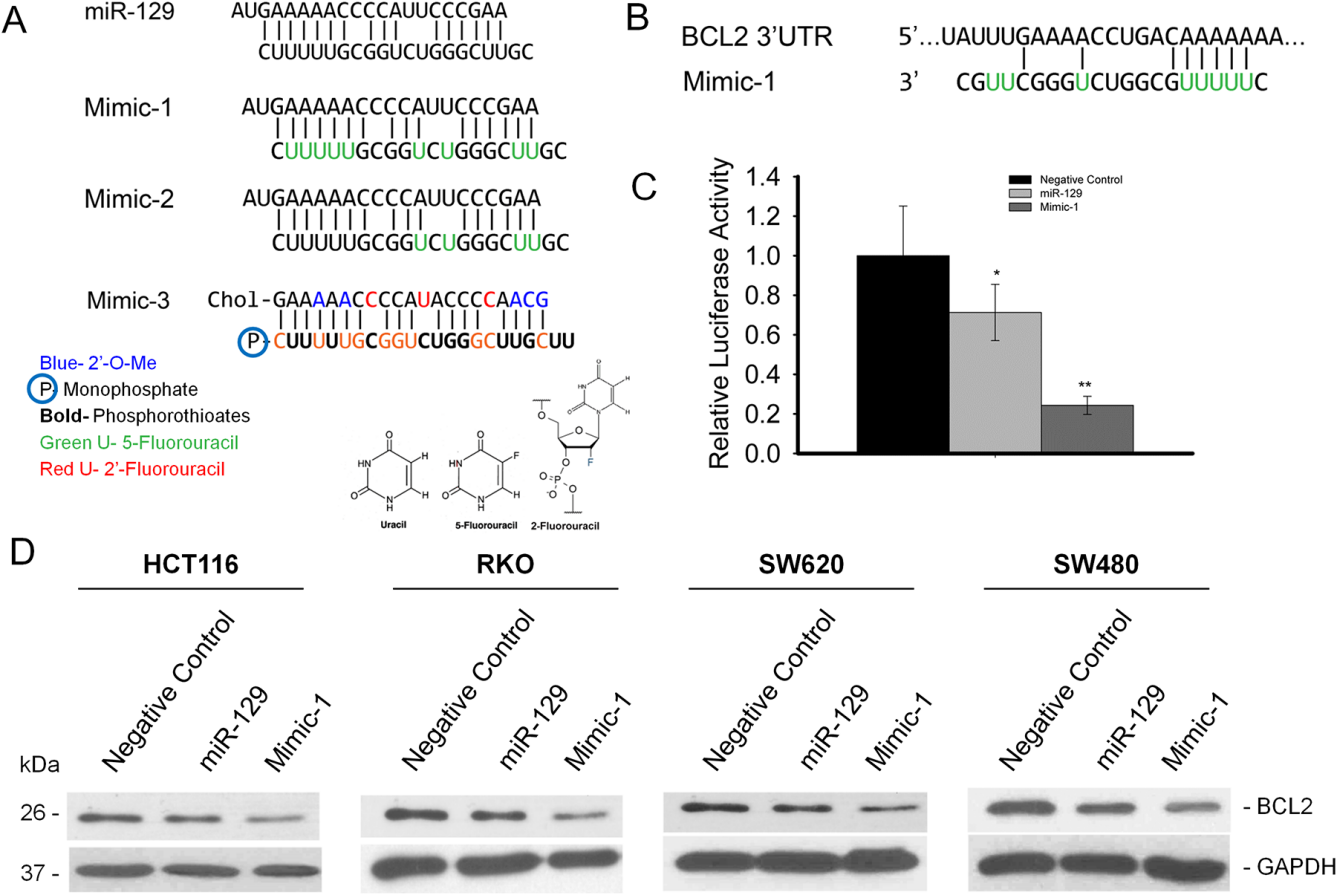
miR-129 mimics retain target specificity in human colon cancer cell lines **(A)** Sequence of miR-129 mimics with various modifications. **(B)** The 3’-UTR binding site of *BCL2* mRNA with miR-129 Mimic-1. **(C)** Transfection of miR-129 or Mimic-1 inhibited firefly luciferase activity of pMIR-REPORT-3’-UTR-BCL2. **(D)** Inhibition of BCL2 expression in HCT116, RKO, SW480, and SW620 colon cancer cell lines analyzed by Western immunoblot. (^*^p<0.05; ^**^p<0.01).

We have designed several different miR-129 mimics to enhance the therapeutic potency without losing target specificity. We have modified the double stranded miR-129 by replacing uracil (U) with 5-FU at different locations (Figure 1A). We have substituted all the U’s with 5-FU in Mimic-1, and we also preserved all the U bases in the seed region and replaced the rest of the U’s with 5-FU in Mimic-2. These modifications were aimed at increasing the therapeutic potency by incorporating the effects of 5-FU into miR-129. Mimic-3 is designed based on a previous report that such modifications are effective in modified miRNA molecules for enhancing miRNA stability. [22] (Figure 1A). Among these modified miR-129 mimics, we found that Mimic-2 and Mimic-3 are as good as the native miR-129 (data not show) in terms of inhibiting cancer cell proliferation and BCL2 expression, while Mimic-1 is the most potent miR-129 mimic at inhibiting colon cancer cell proliferation and target protein expression.

To test the target specificity, we screened all the miR-129 mimics using a luciferase reporter construct containing the miR-129 3’-UTR binding site sequence from *BCL2* mRNA (Figure 1B). Our results show that Mimic-1 has the most potent inhibitory effect on luciferase activity. Mimic-1 reduced luciferase expression by nearly 80% (Figure 1C). To further confirm that Mimic-1 retained target specificity, we transfected Mimic-1 in four different colon cancer cell lines at a concentration of 50 nM. Our results show that Mimic-1 retained target specificity to the known key target, BCL2, via Western immunoblot analysis (Figure 1D).

### miR-129 mimics have potent inhibitory effects on colon cancer cell proliferation

**Figure 2:**
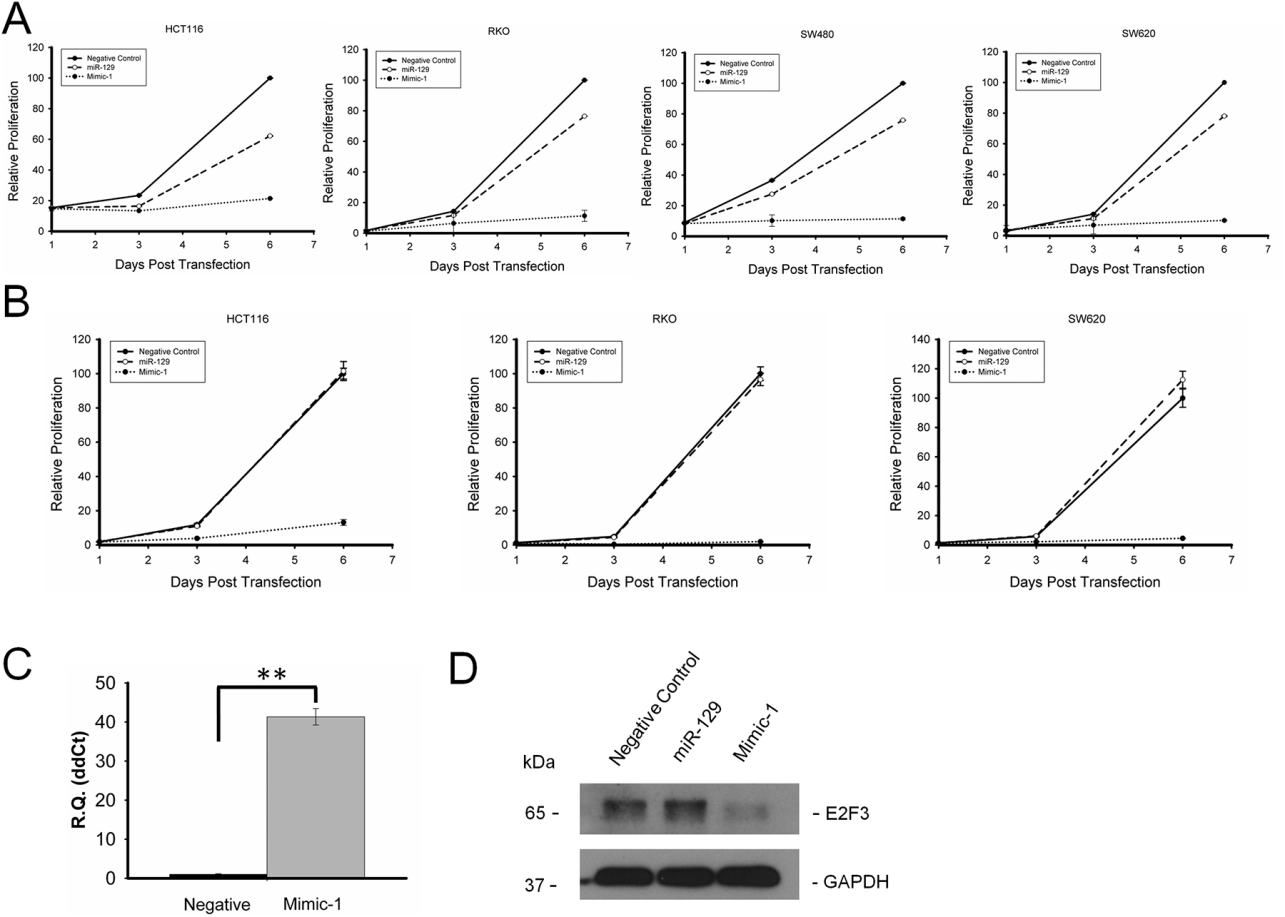
Mimic-1 inhibits colon cancer cell proliferation in HCT116, RKO, SW480, and SW620 colon cancer cell lines with and without transfection reagents **(A)** HCT116, RKO, SW480, and SW620 colon cancer cells were transfected with negative control miRNA, native miR-129, or Mimic-1 at the concentration of 50 nM using oligofectamine, and cell proliferation was measured by WST-1 assay **(B)** HCT116, RKO, SW480, and SW620 colon cancer cells were transfected with negative control miRNA, native miR-129, or Mimic-1 at the concentration of 50 nM without any transfection reagent, and cell proliferation was measured with WST-1 assay. **(C)** qRT-PCR analysis of Mimic-1 levels in colon cancer cells without transfection reagent. **(D)** Without transfection reagent, Mimic-1 expression reduced expression of E2F3. (^**^p<0.01).

We have determined the inhibitory effect of miR-129 mimics on proliferation using 4 different colon cancer cell lines HCT116, RKO, SW480, and SW620. Our results show that Mimic-1 has a profoundly enhanced impact on blocking colon cancer cell proliferation compared to native miR-129. On day 6 post transfection, the cell proliferation of Mimic-1 transfected HCT116, RKO, SW620, SW480 cells were reduced by 78, 88, 88 and 90% of the negative controls, respectively (Figure 2A).

Importantly, we also demonstrated that without using a transfection reagent, we can achieve potent inhibition of cell proliferation with just the naked Mimic-1 (Figure 2B). We also demonstrated that miR-129 can enter cancer cells based on qRT-PCR quantification results (Figure 2C). The reduction of target protein expression also confirmed our finding that naked Mimic-1 can enter the cell and inhibit its target, E2F3 (Figure 2D).

### miR-129 Mimic-1 decreased colon cancer cell viability in a dose dependent manner

**Figure 3:**
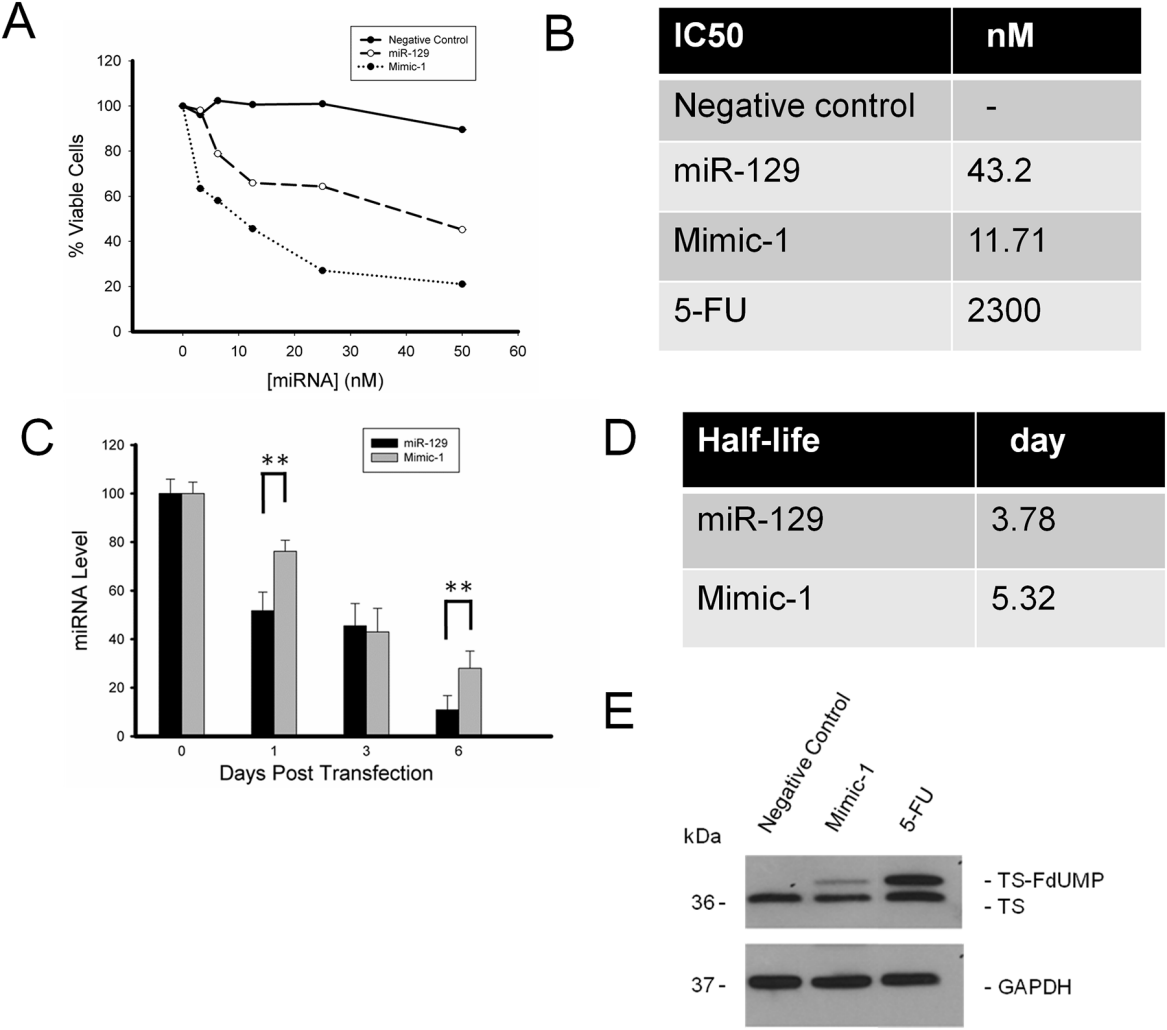
Determination of Mimic-1 half-life and IC^50^ concentration using HCT116 colon cancer cell lines **(A)** Mimic-1 is more potent then unmodified miR-129 on colon cancer cells. **(B)** The IC^50^ for Mimic-1 is 11.71 nM compared to 43.2 nM for miR-129. **(C, D)** Mimic-1 is more stable than miR-129. **(E)** Formation of the FdUMP-TS complex (the upper shift-band) with 5-FU released from the degraded Mimic-1 is shown by Western immunoblot. (^**^p<0.01).

To determine the IC^50^ of miR-129 Mimic-1, HCT116 colon cancer cells were treated with various concentrations of Mimic-1 (Figure 3A). Our results show that the IC^50^ of Mimic-1 is 11.71 nM, while the IC^50^ of native miR-129 is 43.2 nM. The IC^50^ of 5-FU is 2.3 μM (Figure 3B). Mimic-1 is 4-fold more potent than the native miR-129 and over 200-fold more cytotoxic than that of 5-FU.

The stability of Mimic-1 was determined via qRT-PCR analysis using HCT116 colon cancer cells transfected with 50 nM Mimic-1 for 1, 3 and 6 days. Our results show that the expression levels of Mimic-1 decreased slower than that of native miR-129 (Figure 3C). The half-life of Mimic-1 is 5.32 days and the half-life of native miR-129 is 3.78 days (Figure 3D). Despite the enhanced stability of Mimic-1, it does eventually break down releasing 5-FU in to the cell which is metabolized to FdUMP and forms a ternary complex with TS and 5,10-methylenetetrahydrofolate (TS-FdUMP-CH_2_THF) (Figure 3E). Such inhibition of TS activity results in dNTP pool imbalances and causes DNA damage.

### miR-129 mimics induce apoptosis in colon cancer cells

**Figure 4:**
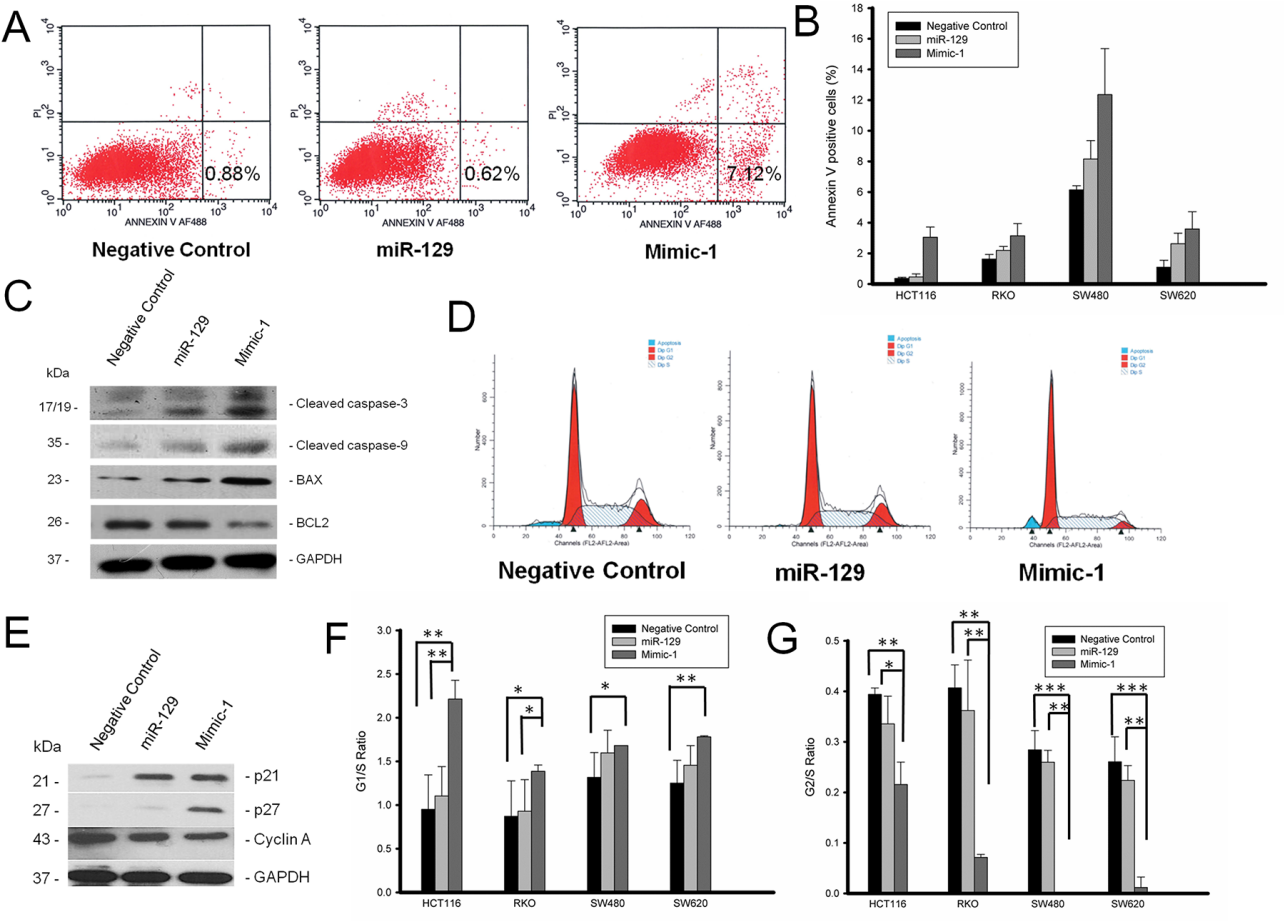
Mimic-1 can induce apoptosis and cell cycle arrest in HCT116, RKO, SW480, and SW620 colon cancer cell lines **(A)** Representative flow cytometry for apoptosis with annexin V and propidium iodide (PI). **(B)** Quantitative analysis of apoptosis in HCT116, RKO, SW480, and SW620 colon cancer cell lines. **(C)** Western immunoblot analysis was performed for activated cleaved caspase-3 and -9 along with BCL2 and BAX expression. **(D)** Representative flow cytometry pattern of cell cycle in HCT116 colon cancer cells treated with Mimic-1. **(E)** Induction of cell cycle regulators p21, p27 and suppression of cyclin A in HCT116 colon cancer cells by Mimic-1. **(F)** Mimic-1 increased G1/S ratio and **(G)** reduced G2/S ratio in HCT116, RKO, SW480, and SW620 colon cancer cell lines. (^*^p<0.05; ^**^p<0.01; ^***^p<0.001).

With *BCL2* being an important target of miR-129, the impact of Mimic-1 on apoptosis was investigated. We quantified cell death using an apoptosis assay in HCT116, RKO, SW480, and SW620 colon cancer cells transfected with negative control miRNA, native miR-129 or Mimic-1. Our results show that Mimic-1 was able to induce apoptosis by 2 to 30-fold in all 4 colon cancer cell lines via a fluorescence-activated cell sorting (FACS)-based FITC–Annexin assay compared to the native miR-129 and negative control miRNA (Figure 4A and 4B).

To determine if the increase in apoptosis is due to the activation of the intrinsic apoptosis pathway, we analyzed the protein expression of cleaved caspase-9 (CASP9) and cleaved caspase-3 (CASP3) by Western immunoblot analysis. Our results show that Mimic-1 treatment activated both caspase-3 and caspase-9 (Figure 4C). In addition, expression of the pro-apoptotic protein, BAX, was also induced by treatment with Mimic-1 in colon cancer cells while the expression of BCL2 was suppressed.

### miR-129 mimics cause cell cycle arrest

As miR-129 suppresses the expression of cell cycle regulator E2F3, the impact of Mimic-1 on cell cycle was determined using flow cytometry analysis. Our results show that Mimic-1 was able to increase G1 arrest while reducing G2 check point control (Figure 4D–4G). The increase in G1 arrest was in part due to the induction of CDKN1A (p21) and CDKN1B (p27) expression along with the reduction of CCNA1 (cyclin A) expression (Figure 4E). In colon cancer SW620 and SW480 cell lines, G2 arrest was completely abolished by Mimic-1 treatment (Figure 4G).

### miR-129 mimics were able to eliminate 5-FU resistant colon cancer stem cells

**Figure 5:**
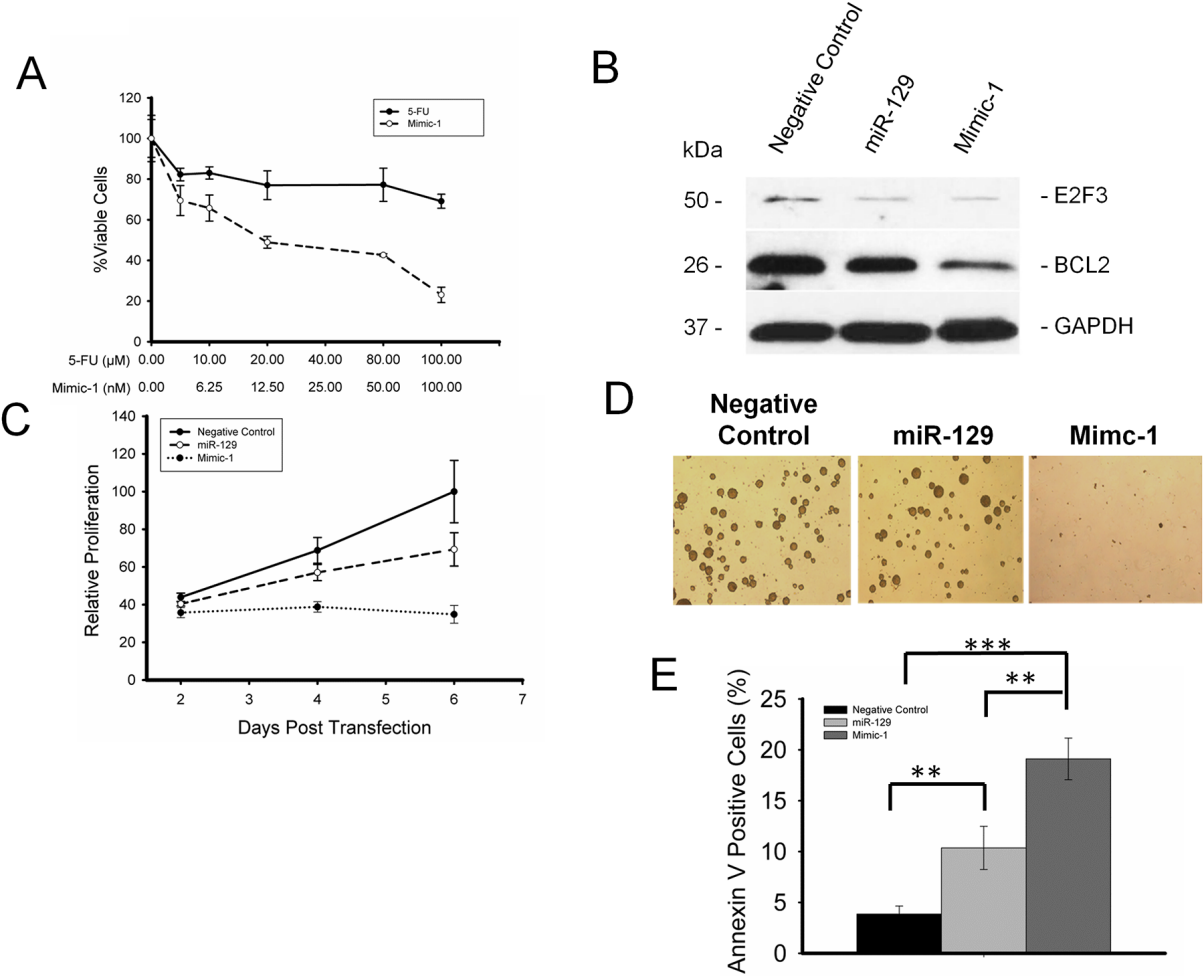
Mimic-1 is effective against chemoresistant colon cancer stem cells (CSCs) **(A)** 100 nM Mimic-1 reduces CSCs viability by 80% while 100 μM 5-FU has minimal effect. **(B)** Mimic-1 reduces the expression of targets E2F3 and BCL2 in colon CSCs. **(C)** Mimic-1 can inhibit proliferation of colon CSCs. **(D)** Mimic-1 inhibits sphere formation in colon CSCs. **(E)** Mimic-1 induces apoptosis in colon CSCs. (^**^p<0.01; ^***^p<0.001).

To determine the impact of Mimic-1 on 5-FU resistant colon cancer stem cells, HCT116 derived colon cancer stem cells were treated with various concentrations of Mimic-1 or 5-FU. Our results show that Mimic-1 was able to eliminate 5-FU resistant colon cancer stem cells by over 80% at 100 nM concentration, while a lethal dose of 5-FU at 100 μM has minimal effect on tumor stem cell viability (Figure 5A).

We further revealed this impact on colon cancer stem cells was due to the inhibition of E2F3, and BCL2 by Western immunoblot analysis (Figure 5B). The inhibitory effect of Mimic-1 on BCL2 expression was stronger than that of native miR-129.

Our results show that Mimic-1 was able to inhibit cell proliferation of HCT116 colon cancer stem cells (Figure 5C). Such inhibitory effect by Mimic-1 was much more potent than native miR-129 as proliferation was nearly completely blocked with 25 nM Mimic-1 on day 6 (Figure 5C). We also demonstrated the impact of Mimic-1 on anchorage independent cell growth using a soft agar assay. Mimic-1 treated colon cancer stem cells formed no visible spheres compared to cells treated with the native miR-129 or control miRNA (Figure 5D).

We further demonstrated that there was a nearly 5-fold increase in apoptosis in colon cancer stem cells treated with Mimic-1 (Figure 5E). Our results clearly show that Mimic-1 can overcome 5-FU resistance in colon cancer stem cells.

### miR-129 mimics inhibit colon cancer metastasis *in vivo*

**Figure 6:**
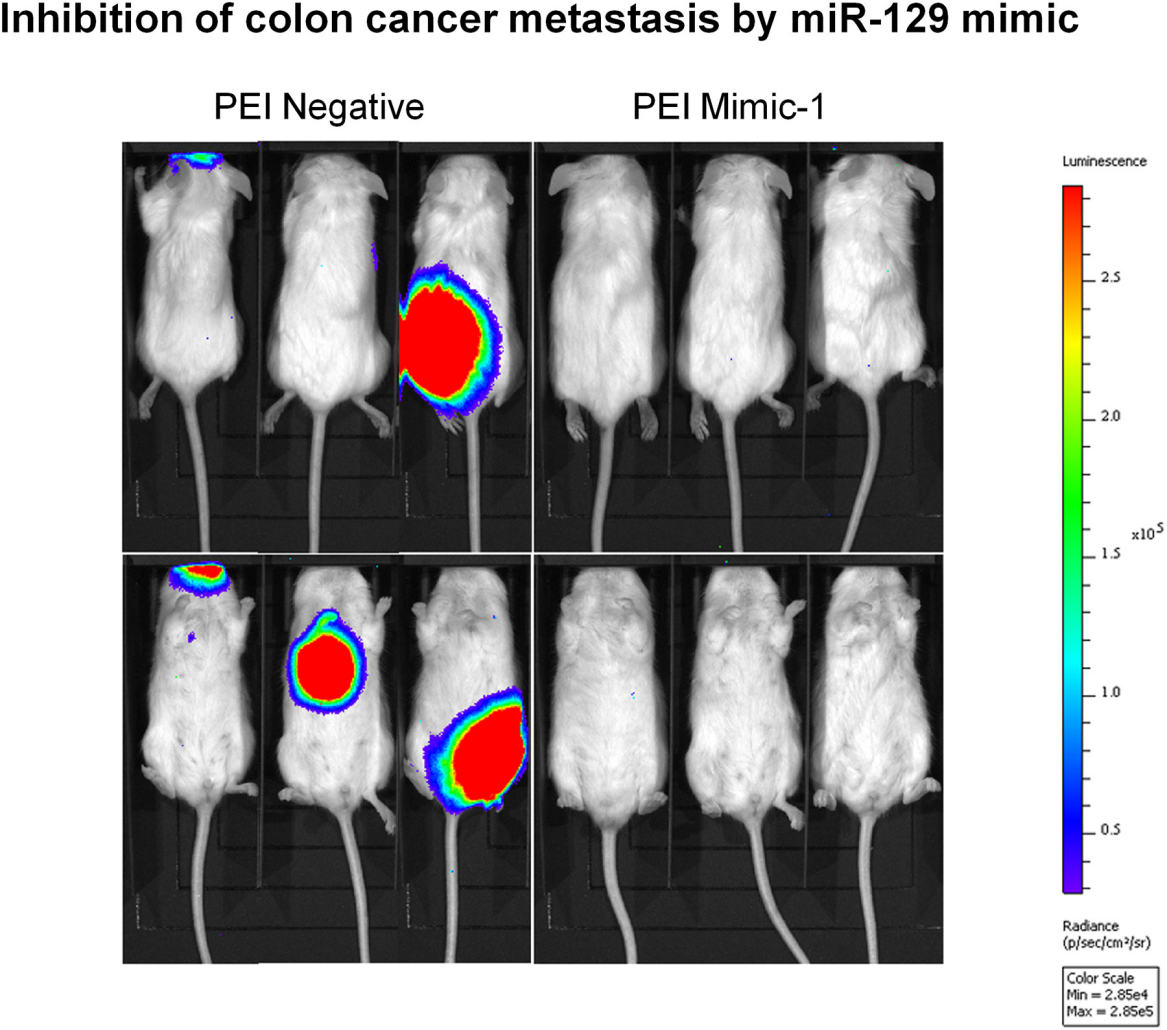
*In vivo* systemic treatment with Mimic-1 inhibits colon cancer metastasis A colon cancer metastasis model was established via tail vein injection of metastatic colon cancer cells. Two weeks after establishing the metastasis, 40 μg of Mimic-1 was delivered by intravenous injection with treatment frequency of one injection every other day for two weeks. Our results show that Mimic-1 was able to inhibit colon cancer metastasis while negative control miRNA has no effect. Mice treated with Mimic-1 have no observed toxicity.

We investigated the impact of Mimic-1 on colon cancer metastasis *in vivo*. A colon cancer metastasis model was established via tail vein injection of metastatic colon cancer cells. Two weeks after establishing the metastasis, 40 μg of Mimic-1 was delivered by intravenous injection with treatment frequency of one injection every other day for two weeks. Our results show that Mimic-1 was able to inhibit colon cancer metastasis while negative control miRNA has no effect (Figure 6). Mice treated with Mimic-1 have no observed toxicity.

## DISCUSSION

Resistance to 5-FU based chemotherapy is the major challenge to the successful outcome of treatment for metastatic colorectal cancer. In this study, we designed and developed a series of novel miR-129 mimics with the goal of enhancing the inhibitory effect of miR-129 on colon cancer and 5-FU resistant colon cancer stem cells. To the best of our knowledge, this is the first time anyone has attempted to integrate the power of two compounds (5-FU and miR-129) into one miRNA entity. The modification is highly innovative as it combines the power of two drugs (miR-129 and 5-FU) uniquely into one as a multi-targeted molecule. We were able to show that this modification did not alter target specificity. Mimic-1 is more potent in inhibiting cell proliferation and its target *E2F3* and *BCL2* compared to native miR-129 (Figure 1-2).

Previous studies from our group have shown that miR-129 is a potent inhibitor in colorectal cancer [19]. We also identified several important targets of miR-129 such as *BCL2, TS*, and *E2F3*. To realize the therapeutic potential of miR-129, in this study, we further modified miR-129 by integrating 5-FU to enhance potency and stability. Our results also show that this modification did not alter target specificity, which is an important feature. The modified miR-129 (Mimic-1) has a longer half-life than native miR-129 (Figure 3C and 3D).

Another important feature of Mimic-1 is that we were able to deliver Mimic-1 without any delivery vehicle (e.g. lipid, dendrimer, viral vector) to cancer cells (Figure 2 B-D). This is a unique and highly innovative feature, as delivery is the major bottleneck of nucleic acid based therapy. We were able to show that Mimic-1 is able to get into the cancer cells to block expression of its targets. We also observed that we can eliminate potential toxicity concerns associated with delivery lipids. The mechanism by which Mimic-1 is able to enter cells without transfection reagents is unclear, however this ability has been demonstrated in locked nucleic acid (LNA) antisense studies [23]. This is the first time that this unique ability has been shown for 5-FU modified miRNA mimics. The mechanism by which the 5-FU modification confers this ability needs to be investigated further.

Mimic-1 was able to induce G1 cell cycle arrest and apoptosis in several colon cancer cell lines by completely abolishing G2 checkpoint control. More importantly, we were able to shown that Mimic-1 can eliminate 5-FU resistant colon cancer stem cells with high potency. Previous studies have shown that the colon cancer stem cells isolated from HCT116 cells express high levels of *PROM1* (CD133), *CD44, EPCAM, SOX2, c-MYC*, and *NANOG* [24]. The isolated colon cancer stem cells are highly resistant to 5-FU treatment [24]. Due to tumor cell heterogeneity, this small fraction of resistant cells are likely the direct cause of chemotherapy resistance in patients. Its ability to eliminate resistant colon cancer stem cells is an ideal feature of Mimic-1 because this is the main issue of 5-FU based chemotherapy. Mimic-1’s ability to target multiple genes, and regulate multiple pathways is a unique advantage for combating the plastic nature of a heterogeneous tumor.

We focused our effort on metastatic colorectal cancer as this is the major hurdle for patients to gain long term survival benefits. Our *in vivo* metastatic mouse tumor model, results show that we can effectively inhibit colon cancer metastasis (Figure 6). Such impact was achieved with the use of *in vivo*-jetPEI. It has been demonstrated in many studies that *in vivo*-jetPEI can effectively deliver nucleic acid based therapeutic agents to multiple organs without any toxicity including immunotoxicity [25]. It can also cross the blood-brain barrier [26]. The well established effectiveness for *in vivo* delivery of miRNA using *in vivo*-jetPEI allowed us to confidently test the therapeutic effectiveness of Mimic-1. In future studies we will investigate the potential for vehicle free delivery of Mimic-1 *in vivo* as we have seen this is effective *in vitro*, though this may present additional challenges as Mimic-1 will need to be stable in circulation and reach target cells. Our results also show that mice treated with Mimic-1 did not show any visible toxic effects such as loss of appetite, or weight and hair loss. Such favorable toxicity profile and potent *in vivo* efficacy support the notion that Mimic-1 may be an ideal candidate for further therapeutic development to treat metastatic colorectal cancer and offer survival benefits to patients with advanced disease.

The miRNA modification strategy can be applied to other miRNAs with therapeutic potential for other tumor types as well. Our study established a solid foundation to expand this approach to the miRNA research field and to impact therapeutic development.

In summary, we have developed a novel and promising miR-129 mimic (Mimic-1) with a number of unique features as a therapeutic candidate for clinical development for treating colorectal cancer, which offers great potential to eliminate resistant colon cancer stem cells. It represents a significant advancement in the development of a nontoxic and highly effective miRNA based cancer therapeutics. Further studies are needed to optimize the *in vivo* delivery, and to systematically determine the pharmacokinetics/pharmacodynamics and *in vivo* toxicity.

## MATERIALS AND METHODS

### miR-129 modifications

miR-129 mimics 1 and 2 were modified by substituting uracil (U) with 5-fluorouracil (5-FU). Mimic-3 was modified with 2’-Flurouracil and Phosphorothioates in the guide strand to enhance stability as well as 2’-O-Methylation of the passenger strand to prevent RISC loading and enhance stability [22]. RNA oligonucleotides with these modifications as well as their corresponding passenger strands were purchased from Dharmacon, (GE Life Sciences). The modified miR-129 and passenger strands were annealed prior to use for transfection (Figure 1).

### Cell lines and transfection

The human colon cancer cell lines HCT116 (wt-p53), RKO, SW480, SW620 were obtained from the American Type Culture Collection (ATCC) and maintained in McCoy’s 5A medium (HCT116), DMEM (RKO, SW480, SW620). Media was supplemented with 10% fetal bovine serum (Thermo Fischer). For Transfection, cells (1×10^5^) were plated in six-well plates and transfected with 50 nM of either mir-129 precursor, non-specific miRNA (Thermo Fischer) or modified miR-129 mimics (Dharmacon, GE Life Sciences) after 24 hours using Oligofectamine (Thermo Fischer) following the manufacturer’s protocols.

Colon cancer stem cells (CSCs) were obtained as described previously [4, 27]. In brief, HCT116 cells were cultured in DMEM/F12 supplemented with B27, 10 ng/mL bFGF, and 20 ng/mL EGF (Life Technologies) in ultra-low attachment flasks. The spheroid cells were maintained by collection through gentle centrifugation, dissociation to single cells and replating. For transfection, CSCs were dissociated and single cell suspensions were plated in ultra low attachment 6 well plates (2×10^5^) cells per well. Transfection was performed using Lipofectamine 2000 (Thermo Fischer) following the manufacturer’s protocols.

To deliver modified miR-129 mimic without transfection reagent, we directly added to cultured colon cancer HCT116 cells (1×10^5^) cells per well, 50 nM of negative control miRNA (Thermo Fischer) or 5-FU modified miR-129 (Mimic-1) (Dharmacon, GE Life Sciences).

### Western immunoblot analysis

Equal amounts of protein (15 μg) extracted from cells lysed in RIPA buffer with protease inhibitor (Sigma) were separated on 10%-12% sodium dodecyl sulfate-polyacrylamide gels by the method of Laemmli [28]. Proteins were probed with anti-TS monoclonal antibody (1:500) (Millipore), anti-BCL2 (1:500) (Invitrogen), anti-E2F3 (1:500) (Santa Cruz Biotech Inc., anti-p21 (1:1000) (Cell Signaling Technologies), anti-p27 (1:500) (Santa Cruz Biotech Inc.), anti-Cyclin A (1:500) (Santa Cruz Biotech Inc.), anti-GAPDH (1:100000) (Santa Cruz Biotech Inc.). Horseradish peroxidase–conjugated antibodies against mouse or rabbit (1:5000, Santa Cruz Biotech Inc.) were used as the secondary antibodies. Protein bands were visualized with autoradiography film using SuperSignal West Pico Chemiluminescent Substrate (Thermo Fischer). Western blot density was quantified using Image J software.

### Cell proliferation and colony formation anlysis

Twenty-four hours after transfection with miRNAs or treatment with naked miRNAs cells were replated in 96 well plates (1000) cells per well. Proliferation was measured on days 1, 3 and 6 post transfection using WST-1 dye (Roche). Cells were incubated with 10 μl of WST-1 dye (per 100 μl of media) for 1 hour and absorbance was read at 450 and 630 nm. The O.D. was calculated by subtracting the absorbance at 630 nm from that at 450 nm. Proliferation experiments were performed three times.

Anchorage-independent proliferation was studied in soft agar assays essentially as described previously (24). CSCs cells were trypsinized and counted and a total of 1×10^5^ cells per well were transfected in 6-well plates with 25 nM Mimic-1 or miR-129 or negative control miRNA with oligofectamine, and 6 hours after transfection, cells were recounted. A total of 20,000 cells in 0.35% agar (Bacto Agar; Becton Dickinson) were layered on top of 1 mL of a solidified 0.6% agar layer in a 35-mm dish. Growth media with B27, 10 ng/mL bFGF, and 20 ng/mL EGF were included in both layers. After 2 weeks of incubation, colonies more than 50 mm in diameter were counted.

### Cell cycle analysis

Twenty-Four hours after transfection, cells were resuspended at 0.5 to 1×10^6^ cells/ml in modified Krishan buffer supplemented with 0.02 mg/ml RNase H (Thermo Fischer) and 0.05 mg/ml propidium iodide (Sigma-Aldrich). Stained cells were detected by flow cytometry and results were analyzed with Modfit LT software. Cell-cycle analysis experiments were performed three times.

### Apoptosis assay

To distinguish between early and late apoptosis, a fluorescein isothiocyanate (FITC)–Annexin assay was done (Becton Dickinson). HCT116, RKO, SW480 and SW620 cells were plated into 6 well plates (1×10^5^) cells per well, after 24h, cells were transfected with 25 nM miRNAs using Oligofectamine. Forty-eight hours after transfection, cells were harvested, stained with propidium iodide and anti-annexin-V antibody (Annexin V-FITC Apoptosis Detection kit, Invitrogen, CA, USA) following the manufacturer’s protocol, and stained cells were detected by flow cytometry. The experiments for the apoptosis assay were performed three times.

### Luciferase assay

To demonstrate the target specificity of Mimic-1, we used a luciferase assay containing a miR-129 binding site. The miR-129 binding sequence in the 3’UTR of *BCL2* mRNA was synthesized with SpeI and PmeI restriction site overhangs (Invitrogen). After annealing, double strand oligonucleotides were inserted into the pMIR-REPORT plasmid (Invitrogen), downstream of the firefly luciferase reporter. The sequences of these synthesized oligonucleotides are: Forward: 5’-CTAGTTCACTGTAGTTTGGTTTTATTTGAAAACCTGACAAAAAAAAAGTTCCAGGT-3’; Reverse: 5’-AAACACCTGGAACTTTTTTTTTGTCAGGTTTTCAAATAAAAC-CAAACTACAGTGA-3’.

Twenty-four hours before transfection, 1.5×10^4^ cells were plated in 96-well plate. 10 nM of miR-129 Mimic-1 or negative miRNA was transfected into cells together with 100ng of pMIR-REPORT-3’-UTR-BCL2 and 1 ng of Renilla luciferase plasmid pRL-SV40 (Promega, Madison, WI, USA) by DharmaFect Duo (Dharmacon, Lafayette, CO, USA) following the manufacturer’s protocol. Luciferase assay was performed 24 hour after transfection by dual-luciferase reporter assay system (Promega). For each sample, firefly luciferase activity was normalized to Renilla luciferase activity and the inhibition by miR-129 was normalized to the control miRNA.

### Real-time qRT-PCR analysis of miR-129 expression

The expression levels of miR-129 in colon cancer cell lines were quantified as previously described [19]. Briefly, the miR-129 specific primer and the internal control RNU44 gene were purchased from Ambion. cDNA synthesis was performed by the High Capacity cDNA Synthesis Kit (Applied Biosystems) with miRNA-specific primers. Real-time qRT-PCR was carried out on an Applied Biosystems 7500 Real-Time PCR machine with miRNA-specific primers by TaqMan Gene Expression Assay (Applied Biosystems). Expression level of miR-129 was calculated by the ddCt method based on the internal control RNU44, normalized to the control group and plotted as relative quantification.

### Cytotoxicity assay

Twenty-four hours after transfection, HCT116 cells were replated in 96-well plates at 2000 cells per well in triplicate in 100μl of medium supplemented with 10% Dialyzed FBS (Thermo Fischer). After 24 hours, fresh medium containing 5-FU alone (ranging from 2 to 7.5 μM) or Mimic-1 alone (ranging from 0 to 100 nM) or Mimic-1 together with 5-FU (at a constant ratio of 1:100, with increasing concentrations of both compounds) were added, and cells were cultured for 72 hours. Cell viability was measured using the WST-1 assay, and concentration-dependent curves were generated based on the cell viability. The IC^50^ for each was calculated using CompuSyn software (www.combosyn.com).

### Mouse colon cancer metastasis models

For the *in vivo* miRNA delivery experiments, we created colon cancer cells that expressed the lenti-luc reporter gene by infecting parental HCT116 cells with a recombinant lentivirus. Luciferase-expressing HCT116 cells (2.0×10^6^ cells per mouse) were suspended in 0.1 mL of PBS solution and injected through the tail vein of each mouse. Two weeks after injection of colon cancer cells, mice were treated via tail vein injection with 40 μg of negative control or Mimic-1 packaged with *in vivo*-jetPEI (Polyplus Transfection). Mice were treated every other day for 2 weeks (8 times). Following treatment, mice were screened using IVIS Spectrum *In vivo* Imaging System (IVIS) (PerkinElmer).

### Statistical analysis

All experiments were repeated at least three times. All statistical analyses were performed with SigmaPlot software. The statistical significance between two groups was determined using Student’s *t*-test (paired *t*-test for clinical samples, and unpaired *t*-test for all other samples). For comparison of more than two groups, one-way ANOVA followed by a Bonferroni-Dunn test was used. Data were expressed as mean ± standard error of the mean (SEM). The statistical significance is either described in figure legends, or indicated with asterisks (^*^p<0.05; ^**^p<0.01; ^***^p<0.001).
